# Pre-exposure prophylaxis use among men who have sex with men who have experienced problematic chemsex

**DOI:** 10.1177/0956462420906927

**Published:** 2020-02-19

**Authors:** Steven Maxwell, Mitzy Gafos, Monty Moncrieff, Maryam Shahmanesh, Oliver Stirrup

**Affiliations:** 1 School of Health in Social Science, University of Edinburgh, Edinburgh, UK; 2 London School of Hygiene and Tropical Medicine, London, UK; 3 London Friend, London, UK; 4 University College London, London, UK

**Keywords:** Men who have sex with men, chemsex, pre-exposure prophylaxis, HIV

## Abstract

Men who have sex with men (MSM) who experience problematic chemsex are at high risk of acquiring HIV due to combined drug use and sexual behaviours. Pre-exposure prophylaxis (PrEP) could substantially reduce the risk of HIV transmission in this group of men. The aim of this study was to examine the biopsychosocial characteristics associated with PrEP use among HIV-negative MSM who have experienced problematic chemsex. This was a cross-sectional analysis of secondary data collected during client assessments at a specialist alcohol and drug service based within the United Kingdom. We compared the socio-demographics, substance use, sexual behaviours and mental health of MSM who reported ever using PrEP to those who reported never using PrEP. Statistical analysis was conducted using the Mann–Whitney U-test for continuous variables and Fisher’s exact test for categorical variables. Between August 2016 and July 2018, 165 HIV-negative MSM who engaged in chemsex had an assessment completed. Thirty-four per cent (n = 50/145) had ever used PrEP. The median age was 36 years (IQR: 30–42), 92% identified as gay (n = 152/165) and 79% were of white ethnicity (n = 130/164). The use of crystal methamphetamine was associated with higher levels of men ever using PrEP (40% versus 21%) (p= 0.047). Men who had ever used PrEP had a higher median number of sexual partners in the previous three months (20 versus 10) (p= 0.004) and had lower level of condom use in their sex lives (median reported 5% versus 50%) (p= 0.010) in comparison to men who had never used PrEP. It is encouraging that men having higher-risk sex had been accessing PrEP. However, further research is required to explore PrEP uptake, retention and adherence in this high-risk group.

## Introduction

There has been growing public health concern about the high risk of HIV transmission posed to men who have sex with men (MSM) who intentionally combine illicit drugs with sex.^1^ Chemsex is the planned use of psychoactive drugs before or during sex to initiate, enhance or facilitate the sexual event.^2^ The drugs associated with chemsex are crystal methamphetamine (crystal meth), mephedrone, gamma-hydroxybutyrate/gamma-butyrolactone (GHB/GBL), ketamine and cocaine.^2,3^ A minority of MSM engage in chemsex but it can involve behaviours which place this group at high risk of HIV acquisition.^4^ The behaviours at one sexual encounter (*chemsex event*) can include multiple sex partners, high rates of condomless anal intercourse (CAI), injecting of drugs and sharing of injecting equipment.^1^

A systematic review found that chemsex behaviours have a negative impact on the psychosocial health and well-being of 14–25% of MSM who engage in the activity.^1^ The increased risk of poor health outcomes for this group of MSM included sexually transmitted infections, HIV, other blood borne viruses, mental ill health and isolation from social supports.^1,4^ This group of men can be described as having experienced ‘problematic chemsex’. This is on the basis that in the substance misuse field, the term ‘problematic use’ is defined as when the substance has had negative-effects on the user’s health and well-being.^5^

Pre-exposure prophylaxis (PrEP) is the use of antiretroviral drugs to lower the risk of HIV acquisition and, if used appropriately, it can reduce the risk of acquisition by over 90%.^6^ The highly efficacious nature of PrEP means that this bio-medical intervention could substantially reduce the risk of HIV transmission for MSM who have engaged in chemsex. Despite this, two systematic reviews found that there was no substantive evidence looking at PrEP use among MSM who had experienced problematic chemsex.^1,7^

The aim of this study was to evaluate the prevalence of PrEP use among MSM who had experienced problematic chemsex and to examine the biopsychosocial characteristics associated with PrEP use. This will help inform the development of evidence-based policy which promotes PrEP uptake within this high-risk population.

## Methods

We conducted a cross-sectional analysis of secondary data which were collected during client assessments by a United Kingdom (UK)-based charity (London Friend) that provides a specialist substance use service. The service is unique in offering specialist harm reduction information and face-to-face support to the lesbian, gay, bisexual and transgender (LGBT) community that are experiencing problematic substance use. The service offers walk-in assessments across six sites in Greater London. LGBT community members self-refer for an assessment and the service is promoted via sexual health clinics, other LGBT services and social media.

The population of interest was HIV-negative MSM over the age of 18 years, who had engaged in chemsex and self-referred to the substance use service. A substance use worker completed an assessment at one interview of the men’s health and social needs by using a structured template (supplementary material 1). All information was self-reported by men at the assessment. Service users were included in the study if at assessment they self-reported: their gender identity as male, the same gender identity as at birth and their HIV status as negative. Service users were identified as having engaged in chemsex if they had reported use of any of the five chemsex-related drugs (crystal meth, GHB/GBL, mephedrone, cocaine and ketamine) and the assessment form had the following questions completed: 1: *a sexual context of drug use (question categories: clubbing, sexual, with friends, on my own and other)*; or 2: *number of partners per chemsex event*. Client assessment information collected between August 2016 and July 2018 was included in the analysis, with the start date corresponding to the first incorporation of information on PrEP into the assessment.

The primary outcome variable for analysis was ‘ever used PrEP’. The assessment form had two separate questions in which men were asked if they were either currently using PrEP and if they had previously used PrEP. To examine the overall level of PrEP use, these two variables were combined to generate the outcome variable. The covariables in the analysis included socio-demographic characteristics, substance use behaviours, sexual health behaviours and mental health factors. The assessment form recorded up to three substances for which clients self-reported problematic use and these were combined to evaluate the overall prevalence of each of the five chemsex drugs. There were varying incomplete levels of data for the variables of interest.

The Mann–Whitney U-test was used for continuous variables and Fisher’s exact test for categorical variables to evaluate associations with PrEP use. All data that were missing were excluded from the analysis. Data analysis was completed using STATA v.15 (StataCorp, College Station, Texas, USA). Project approval was provided in accordance with University College London’s ethics process. The data used for the analysis were anonymised and used in accordance with the relevant data protection legislation.

## Results

Between August 2016 and July 2018, 165 HIV-negative MSM who engaged in chemsex had an assessment completed. Thirty-four per cent (n = 50/145) of the men had ever used PrEP. Twenty-five per cent (n = 36/144) were currently using PrEP, and among the remainder (those who did not report current use), 13% had previously used PrEP (n = 14/109). Ten men who reported no current PrEP use had a missing response for previously using PrEP and were assumed not to have previous use. In addition, one man reported previous use of PrEP without specifying whether this was also current. Table 1 provides a summary of the sample’s socio-demographic characteristics in relation to PrEP use. There was an age range of 21–63 and a median age of 36 years (IQR: 30–42, n = 163). The majority identified as gay (92%, n = 152/165), were of white ethnicity (79%, n = 130/164) and were in regular employment (65%, n = 102/156).

**Table 1.** Summary of socio-demographics.

Demographic type	*n* (%)^a^	PrEP ever	Never PrEP	p-value
Age categories, years (n = 163)				
20–29	35 (22%)	9 (27%)	24 (73%)	0.296
30–39	69 (42%)	20 (33%)	41 (67%)	
40–49	44 (27%)	14 (38%)	23 (62%)	
50–59	13 (8%)	6 (55%)	5 (45%)	
60–69	2 (1%)	1 (100%)	0 (0%)	
Ethnic groups (n = 164)				
Black	11 (7%)	4 (44%)	5 (56%)	0.521
White	130 (79%)	36 (31%)	79 (69%)	
Asian	7 (4%)	4 (67%)	2 (33%)	
Mixed	8 (5%)	2 (33%)	4 (67%)	
Chinese	2 (1%)	1 (50%)	1 (50%)	
Other	6 (4%)	2 (33%)	4 (67%)	
Sexual identity (n = 165)				
Bisexual	6 (9%)	1 (17%)	5 (83%)	0.704
Gay	152 (92%)	48 (35%)	89 (65%)	
Heterosexual	2 (1%)	0 (0%)	0 (0%)	
Queer	2 (1%)	1 (50%)	1 (50%)	
Employment status (n = 156)				
Long term sick/disabled	4 (4%)	0 (0%)	4 (100%)	0.279
Not receiving benefits	2 (1%)	0 (0%)	1 (100%)	
Student	8 (5%)	3 (43%)	4 (57%)	
Regular employment	102 (65%)	36 (38%)	58 (62%)	
Retired	2 (1%)	1 (50%)	1 (50%)	
Unemployed	29 (19%)	5 (20%)	20 (80%)	
Unpaid voluntary work	1 (1%)	0 (0%)	1 (100%)	
Other	8 (5%)	3 (6%)	2 (2%)	

PrEP: pre-exposure prophylaxis.

^a^Per cent and sample number are for the overall sample. Due to non-reported data, the ever/never PrEP figures will not add up to this total.

### Substance use behaviours

**Figure 1.**
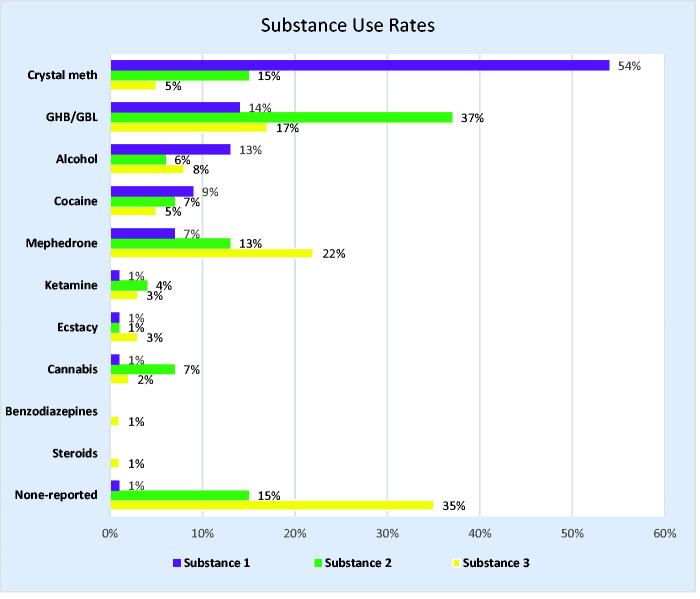
Problem substance 1–3 use rates. GHB/GBL: gamma-hydroxybutyrate/gamma-butyrolactone.

Figure 1 provides a summary of the use rates for problem substances according to the order of reporting by the participant. The most frequently reported primary problem substances were crystal meth (54%, n = 89/164), GHB/GBL (14%, n = 23/164) and alcohol (13%, n = 21/164). In total, 85% (n = 140/164) reported use of a second substance and 66% (n = 108/164) reported a third substance. Table 2 provides a summary of the overall use rates for chemsex drugs and injecting status in relation to PrEP use. The three drugs (crystal meth: 74%, n = 122/164; GHB/GBL: 68%, n = 112/164; mephedrone: 42%, n = 69/164) most commonly associated with chemsex had the highest use rates in the sample. The use of crystal meth was associated with a higher proportion of ‘ever using PrEP’ (40% versus 21%, p = 0.047). A similar relationship with crystal meth was observed for current use of PrEP, although this was not statistically significant (30% versus 13%, p = 0.052). One in three (35%, n = 50/144) were currently injecting and one in five (20%, n= 29/144) had previously injected. There was no statistically significant association between injecting status and ever using PrEP (p= 0.863).

**Table 2.** Chemsex drug use rates and injecting status.

	Never used PrEP	Have used PrEP	p-value
Chemsex drug use^a^			
Crystal meth: 74% (n=122/164)			
Yes	64 (60%)	42 (40%)	0.047
No	30 (79%)	8 (21%)	
GHB/GBL: 68% (n=112/164)			
Yes	62 (61%)	39 (39%)	0.180
No	32 (74%)	11 (26%)	
Mephedrone: 42% (n=69/164)			
Yes	40 (67%)	20 (33%)	0.860
No	54 (64%)	30 (35%)	
Cocaine: 20% (n=33/164)			
Yes	23 (77%)	7 (23%)	0.196
No	71 (62%)	43 (38%)	
Ketamine: 7% (n=12/164)			
Yes	6 (60%)	4 (40%)	0.739
No	88 (66%)	46 (34%)	
Injecting status^a^			
Currently: 35% (n=50/144)	30 (68%)	14 (32%)	0.863
Previously: 20% (n=29/144)	18 (67%)	9 (33%)	
Other people inject me: 1% (n=1/144)	1 (100%)	0 (0%)	
Never: 44% (n=64/144)	35 (61%)	22 (39%)	

GHB/GBL: gamma-hydroxybutyrate/gamma-butyrolactone; PrEP: pre-exposure prophylaxis.

^a^Per cent and sample number for the row variables are for the overall sample. Due to non-reported data, the ever/never PrEP figures will not add up to this total.

### Sexual behaviours

There was a median of three partners (IQR: 1–5) per chemsex event (n = 136/165) and median of ten sexual partners (IQR: 4–20) in the previous three months (n = 146/165). The median number of recent sex partners for men who had used PrEP was 20 (IQR: 9–25) and 10 (IQR: 4–20) for those who never used PrEP (p = 0.004 for difference between groups). There was also a statistically significant difference between men who were currently (20 partners, IQR: 10–30) using PrEP and those not currently (10 partners, IQR: 4–20) using PrEP (p = 0.005). The median percentage level of condom use reported by men within their sex lives was 20% (IQR: 0–80%) (n = 147/165). The median percentage of condom use for men who had used PrEP was 5% (IQR: 0–50%) in comparison to 50% (IQR: 0–90%) for men who had not used PrEP (P = 0.010). In addition, men currently using PrEP (5%: IQR: 0–30%) had lower levels of condom use compared to men who were not currently using PrEP (50%: IQR: 0–80%) (p = 0.021).

### Mental health

Table 3 provides a summary of the responses from the clients’ assessments related to mental health status. One in three (n = 52/165) had a current mental health diagnosis and one in five (n = 31/144) had current contact with mental health services. One in five men (n = 32/148) had previously self-harmed, and previous self-harm was associated with a lower proportion having ever used PrEP (p = 0.002). The majority of the sample did not take psychotropic medication (69%, n = 96/139). There was no significant (p = 0.620) association between ever using PrEP and the current use of psychotropic medication.

**Table 3.** Summary of mental health.

Mental health^a^	Never used PrEP	Have used PrEP	p-value
Mental health diagnosis: 37% (n=52/142)			
Yes	36 (73%)	13 (27%)	0.130
No	48 (59%)	33 (41%)	
Mental health services: 22% (n=31/144)			
Yes	19 (73%)	7 (27%)	0.490
No	67 (64%)	38 (36%)	
Previous suicide attempts: 27% (n=42/154)			
Yes	26 (67%)	13 (33%)	1.000
No	64 (65%)	34 (35%)	
Previous suicidal ideas: 49% (n=75/154)			
Yes	44 (64%	25 (36%)	0.720
No	46 (68%)	22 (32%)	
Current self-harm: 4% (n=6/159)			
Yes	2 (40%)	3 (60%)	0.339
No	90 (67%)	45 (33%)	
Previous self-harm: 22% (n=32/148)			
Yes	26 (90%)	3 (10%)	0.002
No	61 (59%)	42 (41%)	

PrEP: pre-exposure prophylaxis.

^a^Per cent and sample number for the row variables are for the overall sample. Due to non-reported data, the ever/never PrEP figures will not add up to this total.

## Discussion

To our knowledge, this is the first study to look at PrEP use among men who had experienced problematic chemsex. We found that one in three men had ever used PrEP, and these were men who had engaged in higher risk sexual behaviours than men who had never used PrEP. As this population is at high risk of HIV, it is encouraging that a substantial minority had used PrEP.

Among the study sample, one in four were currently using PrEP. In comparison, a systematic review of MSM PrEP use reported a prevalence range of between 1 and 10% (2001–2015), with five studies that collected data in 2015 indicating that one in ten MSM were using PrEP.^7^ However, most of studies included in the systematic review are from heterogeneous MSM samples based in the United States of America (USA). A UK-based study on daily PrEP use reported that 44% of the sample had used chemsex-related drugs prior to study enrolment.^8^ In addition, a study based in France on episodic PrEP use highlighted that 30% of the sample had been under the influence of psychoactive substances during sex.^9^ This study also reported that the correct use of PrEP was associated with periods when sample members were under the influence of psychoactive drugs during sex.^9^

PrEP’s effectiveness is dependent on medication adherence, but this study could not examine this area as these data were not collated. A multi-site PrEP effectiveness trial reported that in the USA there were higher levels of adherence and congruence between self-reported pill taking and blood drug levels than compared to other study sites (Brazil, Andes and Africa/Asia) which had greater levels of disparity between self-reported pill taking and blood drug levels.^10^ A systematic review of MSM PrEP medication adherence in high-income countries reported generally high levels of adherence, but limited evidence suggested drug and alcohol use could contribute towards non-adherence.^7^ One analysis of data from a UK PrEP effectiveness trial reported that there was no association between self-reported adherence and chemsex.^11^ However, purposive research is required to explore the biopsychosocial factors which influence PrEP medication adherence among MSM chemsex participants.

The limited evidence in this study suggests that there are higher levels of PrEP use among MSM who have experienced problematic chemsex. However, as this study sample self-referred to a substance use service, it may suggest they have high levels of awareness into their level of risk-taking behaviours. Further research is required to examine PrEP uptake and retention among this high-risk group.

In this study, it is reassuring that there was higher level of PrEP use among men who had higher levels of sexual partners and lower levels of condom use than compared to men who had never used PrEP. However, among the men who had never used PrEP, half engaged in condomless sex and had high levels of sexual partners. This indicates there is still a need to expand PrEP uptake in this high-risk group. A systematic review of PrEP use identified that a central motivator for PrEP uptake among MSM was the fear of contracting HIV and this may be mediated by the men having multiple sex partners.^7^ Two studies identified that a history of CAI or inconsistent condom use was associated with MSM starting on PrEP.^12,13^ To develop and deliver more effective PrEP uptake initiatives, it would be beneficial to better understand the motivation for PrEP use among MSM chemsex participants.

In the study’s sample, crystal meth was the most commonly reported problematic substance and its use was associated with injecting status. These results are comparable to another UK study which identified in a sample of MSM attending a specialist drug clinic that crystal meth was the most commonly used drug and its use was associated with ever injecting.^14^ In addition, a systematic review on chemsex reported that crystal meth was specifically associated with increased risk of CAI and it commonly featured as a chemsex drug across different regions in high-income countries.^1^

It is important to highlight in our study that the use of crystal meth was associated with a higher proportion of ever using PrEP. It could be speculated that due to the high-risk behaviours linked with crystal meth, users have an awareness of their increased risk of acquiring HIV. To more effectively deliver bespoke PrEP programmes to high-risk chemsex participants, it would be beneficial to understand the dynamic between the various substances used in a sexual context and PrEP use. PrEP has become increasing available in the UK, although with national disparity. Wider evidence suggests there are socio-economic, socio-cultural and stigma-related factors that can act as barriers for accessing PrEP.^7^ As MSM who have experienced problematic chemsex are at high risk of HIV acquisition, it is important we understand how this group can be prioritised to facilitate the expansion of PrEP uptake.

## Strengths and limitations

To our knowledge, this is the first study to examine the biopsychosocial characteristics associated with PrEP use among MSM who have experienced problematic chemsex. However, as the study was cross-sectional it was not possible to establish the direction of association between variables and only provides a ‘snapshot’ of the sample’s behaviours. In addition, the study is limited to a small sample size and did not use statistical mechanisms to correct for confounding factors.

Due to the limited evidence base on MSM who have experienced problematic chemsex, it is difficult to evaluate how representative this sample is of the wider problematic chemsex population. However, this study provides insights into PrEP uptake among this high-risk group. Due to the combined high-risk drug/sexual behaviours and potential consequences, it is fundamental we understand how PrEP can be targeted and used effectively in this high-risk group of men.

## Conclusion

A significant minority of MSM who have experienced problematic chemsex had used PrEP. Men who had used PrEP engaged in higher risk behaviours than men who did not use PrEP, while those who had previously self-harmed were less likely to use PrEP. Comparison with the existing literature suggests there are higher levels of PrEP use in this group of men than the wider MSM population. Further research is required to examine the level of PrEP use, explore factors which facilitate PrEP uptake and evaluate whether there is an inter-relationship between chemsex and PrEP that influences risk behaviours, and retention and adherence to PrEP.

## Supplemental Material

STD906927 Supplemental material - Supplemental material for Pre-exposure prophylaxis use among men who have sex with men who have experienced problematic chemsex Click here for additional data file. Supplemental material, STD906927 Supplemental material for Pre-exposure prophylaxis use among men who have sex with men who have experienced problematic chemsex by Steven Maxwell, Mitzy Gafos, Monty Moncrieff, Maryam Shahmanesh and Oliver Stirrup in International Journal of STD & AIDS
