# Supplementary material for: Pre-exposure prophylaxis use among men who have sex with men who have experienced problematic chemsex
Source: Int J STD AIDS. 2020 Feb 19;31(5):474–80. doi: 10.1177/0956462420906927 (PMC7814093; doi:10.1177/0956462420906927)

ONLINE KEYWORKING REFERRAL FORM

Assessor: ________________ **Date:** ______________

CLIENT DETAILS

FIRST NAME: | **LAST NAME:** | **DATE OF BIRTH:**

ADDRESS:
____
____

POSTCODE: ______________ **BOROUGH:** ______________

EMAIL: ______________

PHONE: ______________

SEX:
☐ Male
☐ Female
☐ Intersex
☐ Non-Binary
☐ Other
If other:

SEXUALITY:
☐ Lesbian
☐ Gay
☐ Bisexual
☐ Queer
☐ Heterosexual
☐ Other

RELIGION:
☐ Buddhist
☐ Christian
☐ Hindu
☐ Jew
☐ Muslim
☐ Sikh
☐ Spiritual
☐ Other
☐ None

GENDER IDENTITY: Different to sex assigned at birth? ☐ Yes ☐ No

NATIONALITY:

DISABILITY / ACCESS REQUIREMENTS: ☐ Yes ☐ No **IF YES, DETAILS:**

ETHNICITY:
☐ White British
☐ White Irish
☐ White Other
☐ Mixed White/Black Caribb.
☐ Mixed White/Black African
☐ Mixed White/Asian
☐ Black Caribbean
☐ Black African
☐ Black Other
☐ Asian Indian
☐ Asian Bangladeshi
☐ Asian Pakistani
☐ Mixed Other
☐ Asian Other
☐ Chinese
☐ Arabic
☐ Other

EMPLOYMENT STATUS:
☐ Regular Employment
☐ Unemployed
☐ Pupil / Student
☐ Long Term Disabled / Sick
☐ Homemaker
☐ Not Receiving Benefits
☐ Unpaid Voluntary Work
☐ Retired From Work
☐ Other

Do you receive sickness / invalidity benefits? ☐ Yes ☐ No

CURRENT ACCOMMODATION:
☐ Private Rented
☐ Staying with Friends / Family
☐ Live on Streets
☐ Own Property
☐ Use Night Hostels
☐ Sleep on Different Friends Floor
☐ Night Winter Shelter
☐ Direct Access Short Stay Hostel
☐ Short Term B&B or Hostel
☐ Squatting
☐ LA or RSL Rented
☐ Approved Premises
☐ Supported Housing / Hostel
☐ Traveller
☐ Settled with Friends / Family
☐ Young Person's Services

PARENTAL STATUS:
☐ Not a Parent
☐ Some of my Children Live with me
☐ All my Children Live with me
☐ None of my Children Live with me

Number of Children under 18 Living in Household:

REFUGEE OR ASYLUM SEEKER? (Please note that this information like any other will be treated confidentially and will only be used anonymously for monitoring services) ☐ Yes ☐ No

REFERRAL DETAILS:

How did you hear about the service?

Have you accessed Antidote previously?
☐ Yes ☐ No If yes, when:

Have you been treated for substance misuse at another organisation?
☐ Yes ☐ No
If yes, please give details:

If yes, do we have permission to liaise with that organisation?
☐ Yes ☐ No

What is your availability for appointments?
☐ Monday Mornings
☐ Thursday Evenings
☐ Other

SUBSTANCE USE

SUBSTANCE	NUMBER IN ORDER OF PRIORITY	DAYS USED LAST MONTH AND AMOUNT	ROUTE OF ADMINISTRATION	AGE OF FIRST USE
Crystal Meth				
GHB/GBL/G				
Mephedrone				
Ketamine				
Cocaine				
Cannabis				
Other				
Alcohol				

INJECTING DETAILS:

Currently injecting/slamming? ☐ Yes ☐ No
Previously injected/slammed? ☐ Yes ☐ No
Sharing needles? ☐ Yes ☐ No
Other people inject for you? ☐ Yes ☐ No

MAIN CONTEXT OF USE:

☐ Sexual ☐ With Friends / Social
☐ Clubbing ☐ On my own ☐ Other

DAILY UNITS OF ALCOHOL:

HEALTH

HIV STATUS:

Are you HIV+? ☐ Yes ☐ No ☐ Not Tested Test Date: ____________

If HIV+:

Are you on medication? ☐ Yes ☐ No
Does drug use interfere with adherence to meds? ☐ Yes ☐ No
Do you attribute your HIV status to substance use? ☐ Yes ☐ No
Did your substance use follow your diagnosis? ☐ Yes ☐ No
Did your substance use escalate after your diagnosis? ☐ Yes ☐ No

If HIV-:

Have you had a course of PEP? ☐ Yes ☐ No How many courses this year?
Did PEP follow a chem sex session? ☐ Yes ☐ No ________
Are you on PrEP? ☐ Yes ☐ No
Have you been on PrEP previously? ☐ Yes ☐ No
Do you consider going on PrEP? ☐ Yes ☐ No
Would you like a sexual health appointment? ☐ Yes ☐ No

SEXUAL LIFE:

Last penetrative sex without a condom (bareback):
Percentage of penetrative sex involving condoms: ____________
Number of different sex partners in past 3 months: ____________
Chems used for sex this year: ☐ Crystal Meth/Tina ☐ GBL/GHB/G ☐ Mephedrone
☐ Cocaine ☐ Other:
Average number of partners per chem sex session: ____________
Last time sober sex without drugs or alcohol: ____________
Use websites or apps to hook up for sex? ☐ Yes ☐ No
Which sites do you prefer? ☐ Grindr ☐ BBRT ☐ Scruff ☐ Hornet ☐ Recon
☐ Gaydar ☐ Manhunt ☐ Other:

SEX WORK:

Are you engaged in sex working or escorting? ☐ Yes ☐ No ☐ Previously
If yes: ☐ Street ☐ Private Premises ☐ Business Premises ☐ Porn Industry

HEP C STATUS:

Are you HEP C+? ☐ Yes ☐ No ☐ Not Tested Test Date:
Previously cleared HEP C? ☐ Yes ☐ No Would you like to arrange a HEP C test? ☐ Yes ☐ No

HEP B STATUS:

Have you been immunised? ☐ Yes ☐ No Total vaccinations you had: ☐ 1 ☐ 2 ☐ 3 ☐ 4
Acquired immunity? ☐ Yes ☐ No Would you like a HEP B intervention? ☐ Yes ☐ No

HPV VACC:

HPV vaccinated? ☐ Yes ☐ No Do you consider getting an HPV vaccination? ☐ Yes ☐ No

antidote @friend LGBT drug and alcohol support

Client Name: ___________________

ASSESSMENT

Client Presentation Notes & Initial Care Plan Notes

GOALS:
☐ Stop ☐ Reduce, then stop ☐ Reduce ☐ Maintain abstinence
☐ Gain control ☐ Harm-reduction ☐ Unsure ☐ Sober sex

Risk Assessment

		Risk Event History (detail each event ticked with a Yes)
**Risk of self-harm or suicide**		
1. Previous suicide attempts?	☐ Yes ☐ No	
2. Previous suicidal ideation?	☐ Yes ☐ No	
3. Current suicide ideation?	☐ Yes ☐ No	
4. Plan made?	☐ Yes ☐ No	
5. Previous self-harm?	☐ Yes ☐ No	
6. Current self-harm?	☐ Yes ☐ No	
**Risk of violence or sexual assault**		
1. Is client at risk of violence?	☐ Yes ☐ No	
2. Is client a risk to others?	☐ Yes ☐ No	
3. Exhibiting aggressive behaviour?	☐ Yes ☐ No	
4. Expressing paranoid delusions?	☐ Yes ☐ No	
**Risk of self-neglect and vulnerability**		
1. Current self-neglect?	☐ Yes ☐ No	
2. Evidence of eating disorder?	☐ Yes ☐ No	
**Risks related to substance use**		
1. Risk of overdose?	☐ Yes ☐ No	
2. Risk of dangerous withdrawals?	☐ Yes ☐ No	
3. Risky sexual practices?	☐ Yes ☐ No	
4. Dangerous injecting practices?	☐ Yes ☐ No	
**Risk to Children**		
1. Contact with children	☐ Yes ☐ No	
2. Is client a main carer?	☐ Yes ☐ No	
3. Use in home with children present?	☐ Yes ☐ No	
4. Is the client pregnant?	☐ Yes ☐ No	

Is the client high risk? ☐ Yes ☐ No

Social services involved? ☐ Yes ☐ No **If yes, details:**

Mental health services involved? ☐ Yes ☐ No **If yes, details:**

Mental health diagnosis? ☐ Yes ☐ No **If yes, details:**

Current Psychotropic Medication?

☐ No ☐ Anti-Depressants ☐ Anti-Psychotics ☐ Anxiolytics/Hypnotics ☐ Other: ___________________

Public Health England

TREATMENT OUTCOMES PROFILE

CLIENT ID ____________

KEYWORKER ____________

SEX
MALE ☐ FEMALE ☐

TREATMENT STAGE
START ☐ REVIEW ☐ EXIT ☐ POST-TREATMENT ☐

DOB DD / MM / YYYY

INTERVIEW DATE DD / MM / YYYY

Use 'NA' only if the client does not disclose information or does not answer

1 SUBSTANCE USE

Record the number of using days in each of the past four weeks, and the average amount used on a using day

	WEEK 4	WEEK 3	WEEK 2	WEEK 1	AVERAGE PER DAY	Total for NDTMS return
A. ALCOHOL	0-7	0-7	0-7	0-7	UNITS	0-28
B. OPIATES/OPIOIDS (ILLICIT) Includes street heroin and any non-prescribed opioid, such as methadone and buprenorphine	0-7	0-7	0-7	0-7	G	0-28
C. CRACK	0-7	0-7	0-7	0-7	G	0-28
D. COCAINE	0-7	0-7	0-7	0-7	G	0-28
E. AMPHETAMINES	0-7	0-7	0-7	0-7	G	0-28
F. CANNABIS	0-7	0-7	0-7	0-7	SPLIFFS	0-28
G. OTHER SUBSTANCE. SPECIFY:	0-7	0-7	0-7	0-7	G	0-28
H. TOBACCO Includes ready-made and hand-rolled cigarettes, cannabis joints with tobacco, cigars, pipe tobacco, shisha/waterpipes, etc	0-7	0-7	0-7	0-7		0-28

2 INJECTING RISK BEHAVIOUR

Record the number of days the client injected non-prescribed drugs during the past four weeks

	WEEK 4	WEEK 3	WEEK 2	WEEK 1	Total for NDTMS return
A. INJECTED	0-7	0-7	0-7	0-7	0-28
B. INJECTED WITH A NEEDLE OR SYRINGE USED BY SOMEBODY ELSE	YES ☐	NO ☐			Y or N (Y if either is yes)
C. INJECTED USING A SPOON, WATER OR FILTER USED BY SOMEBODY ELSE	YES ☐	NO ☐			

3 CRIME

Record the number of days of shoplifting, drug selling and other categories committed during the past four weeks

	WEEK 4	WEEK 3	WEEK 2	WEEK 1	Total for NDTMS return
A. SHOPLIFTING	0-7	0-7	0-7	0-7	0-28
B. SELLING DRUGS	0-7	0-7	0-7	0-7	0-28
C. THEFT FROM OR OF A VEHICLE	YES ☐	NO ☐			Y or N (Y IF EITHER IS YES)
D. OTHER PROPERTY THEFT OR BURGLARY	YES ☐	NO ☐			
E. FRAUD, FORGERY OR HANDLING STOLEN GOODS	YES ☐	NO ☐			
F. COMMITTING ASSAULT OR VIOLENCE	YES ☐	NO ☐			Y or N

4 HEALTH & SOCIAL FUNCTIONING

A. CLIENT'S RATING: PSYCHOLOGICAL HEALTH
(Anxiety, depression, problem emotions and feelings)

0 1 2 3 4 5 6 7 8 9 10 11 12 13 14 15 16 17 18 19 20
POOR — GOOD

Total: 0-20

Record days worked, or at college or school in the past four weeks

	WEEK 4	WEEK 3	WEEK 2	WEEK 1	Total for NDTMS return
B. DAYS IN PAID WORK	0-7	0-7	0-7	0-7	0-28
C. DAYS ATTENDED COLLEGE OR SCHOOL	0-7	0-7	0-7	0-7	0-28

D. CLIENT'S RATING: PHYSICAL HEALTH
(Extent of physical symptoms and bothered by illness)

0 1 2 3 4 5 6 7 8 9 10 11 12 13 14 15 16 17 18 19 20
POOR — GOOD

Total: 0-20

Record accommodation status for the past four weeks

			Total for NDTMS return
E. ACUTE HOUSING PROBLEM	YES ☐	NO ☐	Y or N
F. AT RISK OF EVICTION	YES ☐	NO ☐	Y or N

G. CLIENT'S RATING: OVERALL QUALITY OF LIFE
(Able to enjoy life, gets on with family and partner, etc)

0 1 2 3 4 5 6 7 8 9 10 11 12 13 14 15 16 17 18 19 20
POOR — GOOD

Total: 0-20

CURRENT SITUATION

Your Sense of Control over your Drug/Alcohol Use

**Poor**	**0**	**1**	**2**	**3**	**4**	**5**	**6**	**7**	**8**	**9**	**10**	**11**	**12**	**13**	**14**	**15**	**16**	**17**	**18**	**19**	**20 Good**

Your Sense of Control around Managing Sexual Risk

**Poor**	**0**	**1**	**2**	**3**	**4**	**5**	**6**	**7**	**8**	**9**	**10**	**11**	**12**	**13**	**14**	**15**	**16**	**17**	**18**	**19**	**20 Good**

CLIENT CONSENT FORM

We are committed to protecting your privacy and keeping the information you provide to us confidential. Sometimes we may need to share some information about you with other services involved in your care to better allow us to co-ordinate the treatment and care you receive. If this is the case, we will make every effort to discuss it with you fully first.

Your information will not be used for any other purpose and is managed according to the General Data Protection Regulation (GDPR) and the Data Protection Act 2018. Please note that you have the right to apply for access to any records kept about you; to rectify any incorrect information; and you may withdraw consent for us to process your personal data at any time.

London Friend's Privacy Policy can be viewed at www.londonfriend.org.uk/privacy A worker can provide a hard copy version of this on request.

- I consent to London Friend storing and processing my personal data as long as necessary for the purpose of contacting me and managing my care, treatment or support in accordance with London Friend's Privacy Policy.
- I agree for my personal information to be shared with the services / individuals listed on this form.
- I agree that my anonymised treatment data can be submitted to Public Health England through the National Drug Treatment Monitoring System (NDTMS)
- I agree for information to be used by London Friend to improve and monitor services, including where working in partnerships.
- I understand that I may change or withdraw this consent at any time by emailing London Friend.

CONTACT LIST (please only provide information you consent to us holding)

AGENCY	NAME & CONTACT DETAILS	CONSENT TO CONTACT?
**Emergency Contact**		☐ Yes ☐ No
**GP**		☐ Yes ☐ No
**Social Worker**		☐ Yes ☐ No
**Psychiatrist**		☐ Yes ☐ No
**Other Drugs or Alcohol Agency**		☐ Yes ☐ No
**Community Mental Health Team**		☐ Yes ☐ No
**Family Members**		☐ Yes ☐ No
**NDTMS**	To ensure that treatment services meet the needs of service users, basic information is collected about individuals and the type of treatment you receive. All information is kept strictly confidential and does not include anything which can identify you like your name or address.	☐ Yes ☐ No

**I give consent for correspondence to be sent to my home address:**	☐ Yes ☐ No	**Declining will in no way affect your treatment.**
**I give consent to be put on the mailing list:**	☐ Yes ☐ No	

FEEDBACK

**Do you feel that the work done here today has helped to increase your awareness of issues relating to substance use?**	☐ Yes ☐ No
**Do you feel that the work done here today will help you to improve your overall health & wellbeing?**	☐ Yes ☐ No
**Do you feel your visit here today will improve confidence around sexual health/well-being?**	☐ Yes ☐ No

**DATE**	**SIGNATURE**

AUDIT – C

Questions	Scoring system					Your score
	0	1	2	3	4	
How often do you have a drink containing alcohol?	Never	Monthly or less	2 - 4 times p/month	2 - 3 times p/week	4+ times p/week	
How many units of alcohol do you drink on a typical day when you are drinking?	1 -2	3 - 4	5 - 6	7 - 9	10+	
How often have you had 6 or more units if female, or 8 or more if male, on a single occasion in the last year?	Never	Less than monthly	Monthly	Weekly	Daily/almost daily	

Scoring: A total of 5+ indicates increasing or higher risk drinking.
An overall total score of 5 or above is AUDIT-C positive.

SCORE

Remaining AUDIT questions

Questions	Scoring system					Your score
	0	1	2	3	4	
How often during the last year have you found that you were not able to stop drinking once you had started?	Never	Less than monthly	Monthly	Weekly	Daily/ almost daily	
How often during the last year have you failed to do what was normally expected from you because of your drinking?	Never	Less than monthly	Monthly	Weekly	Daily/ almost daily	
How often during the last year have you needed an alcoholic drink in the morning to get going after a heavy drinking session?	Never	Less than monthly	Monthly	Weekly	Daily/ almost daily	
How often during the last year have you had a feeling of guilt/remorse after drinking?	Never	Less than monthly	Monthly	Weekly	Daily/ almost daily	
How often during last year have you been unable to remember what happened the night before because you'd been drinking?	Never	Less than monthly	Monthly	Weekly	Daily/ almost daily	
Have you or somebody else been injured as a result of your drinking?	No		Yes, but not in last year		Yes, during last year	
Has a relative or friend, doctor or other health worker been concerned about your drinking or suggested that you cut down?	No		Yes, but not in last year		Yes, during last year	

SCORE

Scoring: 0 – 7 Lower risk, 8 – 15 Increasing risk, 16 – 19 Higher risk, 20+ Possible dependence

TOTAL

This is one unit of alcohol…

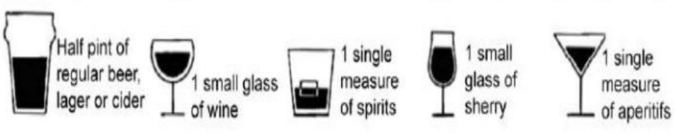

…and each of these is more than one unit

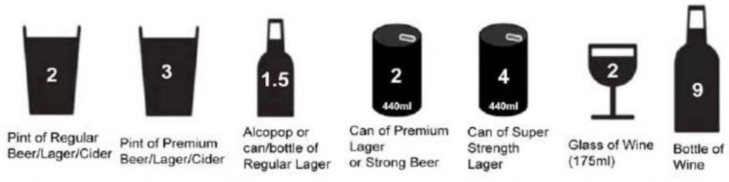

Supplement: STD906927 Supplemental material - Supplemental material for Pre-exposure prophylaxis use among men who have sex with men who have experienced problematic chemsex [file STD906927_Supplemental_material.pdf]
